# Targeting Proteotoxic Stress in Cancer: A Review of the Role that Protein Quality Control Pathways Play in Oncogenesis

**DOI:** 10.3390/cancers11010066

**Published:** 2019-01-09

**Authors:** Matthew Ho Zhi Guang, Emma L. Kavanagh, Luke Paul Dunne, Paul Dowling, Li Zhang, Sinéad Lindsay, Despina Bazou, Chia Yin Goh, Cathal Hanley, Giada Bianchi, Kenneth C. Anderson, Peter O’Gorman, Amanda McCann

**Affiliations:** 1 UCD Conway Institute of Biomolecular and Biomedical Science, Dublin, Dublin 4, Ireland; 11100787@ucdconnect.ie (M.H.Z.G.); Emma.Kavanagh@ucdconnect.ie (E.L.K.); sinead.lindsay@ucd.ie (S.L.); chia.goh@ucdconnect.ie (C.Y.G.); 2 UCD School of Medicine, College of Health and Agricultural Sciences, University College Dublin, Belfield Dublin, Dublin 4, Ireland; cathal.hanley@ucdconnect.ie; 3 LeBow Institute for Myeloma Therapeutics and Jerome Lipper Multiple Myeloma Center, Department of Medical Oncology, Dana Farber Cancer Institute, Harvard Medical School, Boston, MA 02115, USA; luke.dunne.2015@mumail.ie (L.P.D.); giada_bianchi@dfci.harvard.edu (G.B.); kenneth_anderson@dfci.harvard.edu (K.C.A.); 4 Biology Department, National University of Ireland Maynooth, Co. Kildare W23 F2K8, Ireland; paul.dowling@nuim.ie; 5 Department of Hematology, Sichuan University, Chengdu, Sichuan 610041, China; drzhangli2014@sina.com; 6 Haematology Department, Mater Misericordiae University Hospital, Dublin Dublin 7, Ireland; despinabazou@gmai.com

**Keywords:** proteotoxic stress, chemoresistance, proteasome, unfolded protein response, autophagy, multiple myeloma, triple negative breast cancer, protein quality control

## Abstract

Despite significant advances in cancer diagnostics and therapeutics the majority of cancer unfortunately remains incurable, which has led to continued research to better understand its exceptionally diverse biology. As a result of genomic instability, cancer cells typically have elevated proteotoxic stress. Recent appreciation of this functional link between the two secondary hallmarks of cancer: aneuploidy (oxidative stress) and proteotoxic stress, has therefore led to the development of new anticancer therapies targeting this emerging “Achilles heel” of malignancy. This review highlights the importance of managing proteotoxic stress for cancer cell survival and provides an overview of the integral role proteostasis pathways play in the maintenance of protein homeostasis. We further review the efforts undertaken to exploit proteotoxic stress in multiple myeloma (as an example of a hematologic malignancy) and triple negative breast cancer (as an example of a solid tumor), and give examples of: (1) FDA-approved therapies in routine clinical use; and (2) promising therapies currently in clinical trials. Finally, we provide new insights gleaned from the use of emerging technologies to disrupt the protein secretory pathway and repurpose E3 ligases to achieve targeted protein degradation.

## 1. Introduction

### 1.1. Tight Regulation Over the Central Dogma of Molecular Biology is Essential for Cell Survival

The key to cell survival and function is the tight control over the central dogma of molecular biology. This is the structured flow of genetic information from DNA → mRNA → protein that underlies the dynamic processes in living cells. Due to the fact that protein homeostasis is absolutely crucial to cell survival, it is tightly regulated at different stages of the DNA-mRNA-protein pathway: (1) transcription (through epigenetic mechanisms), (2) RNA metabolism and processing, (3) ribosomal protein synthesis, (4) protein folding (aided by chaperones), (5) protein translocation, (6) protein assembly/disassembly, and (7) protein clearance [1,2]. Accumulation of misfolded proteins, a consequence of disrupted protein homeostasis, initiates endoplasmic reticulum (ER) stress that, if not promptly managed, leads to a global decline in cellular function and cell death [1].

### 1.2. Proteotoxic Stress: A Secondary Hallmark of Cancer

The protein synthesis process is intrinsically prone to errors. It has been estimated that up to 30% of newly synthesized proteins are degraded by the proteasome within minutes of protein translation in mammalian cells [3]. These rapidly degraded proteins are called defective ribosomal proteins (DRiPs) or rapidly degraded polypeptides (RDPs), and if not removed, can dramatically increase basal proteasome load and cell stress [4]. Cancer cells generally synthesize proteins (and therefore DRiPs) more rapidly than normal cells due to increased cell division coupled to cell growth [5]. For example, cancer cells that over-activate mTORC1, which promotes protein synthesis through inhibition of 4E-BPs and activation of S6K1, become reliant on the immunoproteasome to prevent the accumulation of misfolded proteins resulting from mTORC1 activation [6,7]. Such is the importance of proteostasis, that mutations in RAS, PTEN, TSC1, and mTORC1 itself, enhance the formation of immunoproteasomes as a mechanism to cope with increased proteotoxic stress resulting from downstream oncogenic processes [7]. Besides DRiPs, genetic (exemplified by structural mutations) and environmental factors such as hypoxia, oxidative stress, and nutrient deprivation are key activators of the integrated stress response (ISR), a cytoprotective response to proteotoxic stress [4,8,9].

### 1.3. Endoplasmic Reticulum (ER) Stress is Closely Linked to Oxidative Stress in Cancer

Proteotoxicity is a key feature of both oxidative and reductive stress [10,11,12]. ER protein-folding homeostasis can be disrupted by altered redox balance within the ER lumen which disrupts protein folding to cause ER stress [13]. Accumulating evidence shows that ER stress signalling is elicited in response to treatments that enhance the intracellular release of reactive oxidative species (ROS) [14,15,16]. On the other hand, oxidative protein folding (disulfide bond formation) in the ER, results in the release of ROS as a by-product, which can then be used to activate a variety of transcription factors including NF-κB, AP-1, p53, HIF-1α, PPAR-γ, β-catenin/Wnt, and Nrf2 to help cancer cells maintain their high proliferation rate [17,18]. While moderate increases in ROS support tumorigenesis, excessive levels of oxidative stress causes damage to cancer cells; a feature that can be exploited therapeutically using ROS-modulating agents [19].

### 1.4. Aneuploidy Contributes to Proteotoxic Stress

Aneuploidy, which in turn is a manifestation of genomic instability, contributes to enhanced and elevated proteotoxic stress in cancer cells [20,21,22]. Recent studies performed in aneuploid yeast have shown that excessive protein production, secondary to extra chromosomes, disrupts proteostasis resulting in growth inhibition [23,24]. In human cancer cell lines, polyploidy has been associated with the induction of the unfolded protein response (UPR) and autophagy [25].

Perhaps the best example of the proteotoxic stress phenotype is multiple myeloma (MM), a type of plasma cell dyscrasia typified by near universal aneuploidy (and genomic instability), and characterised by high synthesis rates of immunoglobulins, and therefore DRiPs [26,27]. MM cells exhibit stigmata of ongoing proteotoxic stress with the accumulation of polyubiquitinated proteins, the baseline induction of the UPR, and significant reliance on proteostasis pathways for survival [27,28,29].

## 2. Overview of the Protein Quality Control System

**Figure 1:**
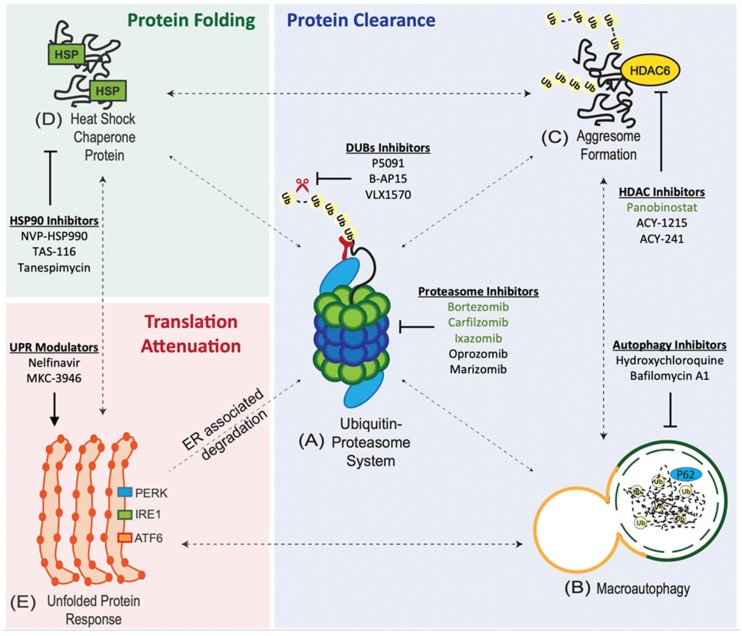
Protein handling pathways in cancer cells. cancer cells have to cope with a large burden of misfolded proteins, which if not managed appropriately results in endoplasmic reticulum (ER) stress and eventual cell death. As such, cancer cells are highly dependent on a tightly regulated network of protein quality control pathways such as (**A**) the ubiquitin proteasome system (UPS), (**B**) macroautophagy, (**C**) aggresome formation, (**D**) heat shock response, and (**E**) the unfolded protein response.

Cancer cells maintain the integrity of the proteome through an interconnected network of proteostasis pathways (Figure 1). At its core is the ubiquitin proteasome system (UPS), which works together with the macroautophagy (autophagy-lysosome) system and the aggresome pathway to regulate protein clearance [20]. The unfolded protein response (UPR) is an adaptive response to ER stress that, in addition to regulating ribosomal protein synthesis, is also able to recruit the other proteostasis pathways (such as the aggresomal or macroautophagy pathways) to either maintain proteostasis or induce apoptosis if ER stress remains unmitigated [30]. Upstream of the proteasome, the heat shock chaperone protein system, which can be induced in response to proteotoxic stress, contributes to protein homeostasis by regulating protein folding [31].

### 2.1. Ubiquitin Proteasome System (UPS)

Protein degradation through the UPS begins with the polyubiquitination of targeted (misfolded) proteins by a three-enzyme cascade involving E1 (activating), E2 (conjugating), and E3 (ligating) enzymes (Figure 1A) [32]. Polyubiquitinated proteins are subsequently translocated to the 26S proteasome; an ATP-dependent multi-catalytic complex comprising a 20S catalytic core flank on either ends by 19S regulatory caps [33]. Polyubiquitinated proteins are recognized by the 19S regulatory substrate and deubiquitinating enzymes (RPH11, UCH37, and USP14) within the 19S cap [34]. The 19S cap facilitates the removal of the polyubiquitin chain, which would otherwise sterically hinder the translocation of misfolded proteins through the narrow pore formed by the 19S cap into the catalytic core [34]. The 20S core contains three main catalytic subunits: the β1 (caspase-like activity), β2 (trypsin-like activity), and β5 (chymotrypsin-like activity) subunits [35]. The proteasome inhibitors (PIs) bortezomib, carfilzomib, ixazomib, and oprozomib largely target the β5 subunit while the pan-proteasome inhibitor marizomib has been shown to inhibit all three β subunits [36,37,38,39].

De-ubiquitinating enzyme (DUB) inhibitors (P5091, B-AP15, and VLX1570), on the other hand, prevent the removal of polyubiquitin chains, resulting in the accumulation of misfolded proteins and apoptosis, without inhibiting the catalytic subunits of the proteasome [40,41,42]. DUB inhibitors could potentially have clinical utility in cases of bortezomib resistance mediated by mutations in the proteasomal catalytic subunits [43,44].

Recent research has identified the compensatory proteasome “bounce-back” response, mediated by NRF1 activation as a potential mechanism of resistance to proteasome inhibition in cancer cells [45]. Nuclear factor erythroid-derived 2-related factor 1 (NRF1) is an ER-resident transcription factor of the cap “*n*” collar basic leucine zipper family that is continually retro-translocated and degraded by the proteasome [45]. When proteasome activity is inhibited, the transcription factor NRF1 (nuclear factor erythroid derived 2-related factor 1) escapes degradation and is cleaved and activated by the aspartyl protease DDI2 (DNA-damage inducible 1 homolog 2) [46]. Activation of NRF1 results in the transcription of proteasome (PSM) genes followed by de novo proteasome formation [45]. In contrast with NRF1 WT cells, NRF1 biallelic knockout cells lacked the ability to recover proteasome activity in response to proteasome inhibition [45].

### 2.2. Macroautophagy (Autophagy-Lysosome System)

Macroautophagy is a lysosomal degradation pathway that plays an important role in proteostasis through the sequestration and removal of misfolded proteins (Figure 1B) [47]. This affords cancer cells the flexibility to tolerate stress, particularly when the other proteostasis pathways are overwhelmed. Misfolded proteins are engulfed by autophagosomes and upon fusion with lysosomes, are degraded by lysosomal hydrolases [48]. While the scientific community remains divided as to whether macroautophagy plays a protective or deleterious role in cancer, the general consensus is that depending on the type of cancer, a basal level of autophagy provides an alternative proteolytic pathway and might be essential for survival especially in times of proteotoxic stress [47]. However, persistent and uncontrolled proteotoxic stress induces autophagic cell death through the over-expression of Beclin-1 (an autophagy regulatory protein) [47,49]. This highlights a common theme that proteostasis is a delicate balance that needs to be struck through (1) precise regulation within each protein quality control pathway, and (2) tightly-controlled interactions between the different proteostasis pathways (Figure 1).

### 2.3. Aggresome Pathway

Polyubiquitinated (misfolded) proteins that coalesce to form aggresomal particles are transported towards the microtubule organizing center (MTOC) in a histone deacetylase 6 (HDAC6)-dependent fashion and sequestered into aggresomes that target these proteins for degradation by autophagy, or refolding by the heat-shock protein (HSP) chaperone system (Figure 1C) [50].

### 2.4. Heat-Shock Protein (HSP) Chaperone System

As eluded to previously, misfolded proteins can undergo one of two fates: (1) protein clearance through the aforementioned pathways or (2) protein refolding with the help of HSP chaperones. HSPs are a large family of chaperones that play an important role in protein folding, particularly in the presence of hypoxia, and oxidative and thermal stress (Figure 1D) [51]. Compared to normal cells, cancer is even more reliant on the HSP chaperone machinery for proliferation and survival because (1) cancer oncoproteins are often misfolded, and (2) the high levels of DRiPs production [51]. The two most widely studied HSPs in cancer are HSP70 and HSP90, both of which have been found to stabilize dominant-negative (inactivating) mutant p53, thereby allowing cancer cells to evade anti-growth signals [52]. HSP70 and HSP27, on the other hand, are able to directly interact with protein intermediates in apoptosis pathways, thereby inhibiting programmed cell death [52]. Recent studies have also found that HSP90 protects telomeres from erosion, thereby contributing to the limitless proliferation and avoidance of senescence in cancer cells [52]. Furthermore, HSPs have been implicated in all aspects of the various hallmarks of cancer such as angiogenesis, tumor cell invasion and metastasis, tumor progression, and drug resistance [52]. 

HSP90, is also involved in chaperone-mediated autophagy (CMA), a selective form of autophagy involving the recognition of specific targeting motifs in substrates (by cytosolic chaperones) and delivery to lysosomes [53]. Specifically, HSP90 interacts with the CMA substrate-chaperone complex at the lysosomal membrane and pharmacologic inhibition of HSP90 reduces CMA activity [53]. Lastly, HSPs also play a key role in regulating the unfolded protein response. BiP/GRP78, a HSP70 superfamily protein is a major ER chaperone protein that serves as the master regulator of ER protein quality control by controlling the activation of the ER-transmembrane signalling molecules [54,55].

Recently, there has been growing interest in developing inhibitors against heat shock factor 1 (HSF1), the “master regulator” of the heat shock response, in an attempt to avoid compensatory upregulation of individual chaperones [56]. While the previous view was that HSF1’s main impact on tumor biology occurs indirectly through the regulation of HSP90 and HSP70, recent research has shown that HSF1 may play a more direct role in altering the transcriptome of cancer cells [57].

### 2.5. The Integrated Stress Response (ISR)

The ISR is a common adaptive pathway that is activated in response to a variety of stress signals; both extrinsically (such as hypoxia, nutrient deprivation, viral infection), and intrinsically (such as ER stress) [58,59,60,61,62]. Cellular stress signals activate four distinct serine/threonine protein kinases: GCN2 (nutrient deprivation), PKR (viral infection), HRI (heme deprivation), and PERK (ER stress) that converge on the phosphorylation of eIF2α (the core of ISR) [9,63]. Phosphorylation of eIF2α results in the global attenuation of Cap-dependent mRNA translation coupled with the preferential translation of ISR-specific mRNAs such as ATF4 and the expression of ATF4 target genes that alleviate proteotoxic stress [9]. As part of a negative feedback loop, GADD34, which is induced by the ISR, dephosphorylates eIF2α to terminate the ISR and restore protein synthesis and cell normal cell function [64]. While the other ISR regulators such as GCN2 and HRI have also been linked to cancer survival and proliferation, this review will focus on the role that PERK and the unfolded protein response (UPR) play in response to ER protein misfolding and proteotoxic stress [65,66].

### 2.6. Endoplasmic Stress and the Unfolded Protein Response (UPR)

Central to the protein quality control mechanism of the cell, is a process termed ER-associated degradation (ERAD), by which improperly folded proteins are retained in the ER and delivered for proteasome degradation after retro-translocation into the cytosol [30]. The cytosolic ATPase p97 (VCP/Cdc48) delivers ubiquitinated proteins from the ER to the proteasome by translating ATP hydrolysis into mechanical force; thereby playing a crucial role in ERAD [67]. Due to the fact that protein degradation is coupled via the UPS, to the dislocation of proteins from the ER into the cytosol (a key step of ERAD), any conditions blocking ER retro-translocation and/or the UPS may also result in misfolded protein accumulation within the ER [68,69,70]. Therefore, misfolded proteins trigger the UPR regardless of whether they accumulate within or outside the ER (such as in the nucleus or cytosol) [71]. When the misfolded protein load within the ER exceeds the threshold of ERAD, the resultant accumulation of misfolded proteins causes endoplasmic stress and induces the UPR through the activation of the stress sensors, (1) activating transcription factor 6 (ATF6), (2) inositol-requiring enzyme 1 (IRE1), and (3) protein kinase RNA-like ER kinase (PERK), which represent the three branches of the UPR (Figure 1E) [30]. The three branches of the UPR operate in parallel as feedback loops that mitigate ER stress. Activation of PERK (and downstream phosphorylation of eIF2α) and IRE1 (and downstream splicing of XBP1 mRNA), regulates ER expansion and decreases global protein synthesis to decrease the flux of proteins entering the ER [30]. ATF6 activation leads to the upregulation of ER-resident chaperone proteins involved in protein folding (such as BiP, protein disulphide isomerase, GRP94) to ultimately increase ER protein folding capacity [30]. On the other hand, when homeostasis fails (due to prolonged and overwhelming stress), prolonged activation of the PERK-ATF4 pathway activates the transcription factor C/EBP homologous protein (CHOP) which triggers apoptosis, outlining the double-edged nature of the UPR [30].

Studies have shown that crosstalk exists between the UPS, autophagy, and the UPR [72,73,74]. Specifically, activation of the PERK-eIF2α pathway and IRE1 has been implicated in the activation of autophagy with ATF4 and CHOP found to transcriptionally regulate multiple autophagy-related (ATG) genes [75]. Furthermore, the cytoplasmic portion of IRE1 is known to bind TNF receptor-associated factor 2 (TRAF2) which, through its kinase activity, couples ER stress to c-Jun N-terminal kinase (JNK) activation [76]. Activation of JNK leads to Bcl-2 phosphorylation, allowing Beclin-1 dissociation and activation of the Phosphoinositide-3-Kinase (PI3K) complex and autophagy [77].

Activation of the UPR has been implicated in bortezomib resistance. Specifically, IRE1α initiates the splicing of X-box binding protein 1 (Xbp1) [78,79], which is an important transcription factor involved in the terminal differentiation of B-lymphocytes to plasma cells and subsequent induction of antibody secretion [80]. Indeed, mature MM cells that have higher Xbp1s expression and higher levels of antibody secretion have been found to be more sensitive to bortezomib compared to immature Xbp1s(-) MM cells arrested at the pre-plasmablast stage [81]. On the other hand, mutations resulting in Xbp1 inactivation have been found in two treatment-refractory MM patients [81,82]. A likely explanation for this is that terminal plasma cell differentiation results in (1) progressively impaired proteasomal capacity, and (2) increased antibody secretion resulting in a proteasome workload-capacity imbalance; thereby, sensitizing cells to apoptosis by proteasome inhibition [4,83].

## 3. Exploiting Proteotoxic Stress in Hematologic Malignancies: Multiple Myeloma

### 3.1. Proteasome Inhibitors (PIs)

Proteasome inhibition has emerged as an extremely effective targeted therapeutic strategy as it exploits the unique biology of MM in that myeloma cells have to deal with large amounts of misfolded proteins, and hence, high levels of proteotoxic stress due to extensive immunoglobulin synthesis (Table 1) [84,85]. Since the first phase I bortezomib trials almost 15 years ago, proteasome inhibitors (PIs) have become a mainstay of therapy, contributing substantially to the increase in overall survival of patients diagnosed with myeloma over the years [86]. PIs in clinical use can be classified into three groups: (1) boronates, (2) epoxyketones, and (3) γ-lactam-β-lactones (salinosporamide) based on their chemical structure and active moiety. Bortezomib is the “first-in-class” boronic acid PI that reversibly inhibits the chymotrypsin-like activity of the proteasome [87]. Bortezomib first received FDA approval in 2003 and has since been seen as a major break-through in the treatment of MM [88]. A meta-analysis of 16 studies involving 5626 patients with MM reported that bortezomib prolongs overall survival (OS), progression free survival (PFS), and improves response rates in trials of bortezomib versus no bortezomib with the same or different background therapy [89].

Carfilzomib and oprozomib, on the other hand, are irreversible chymotrypsin-like, epoxyketone PIs while ixazomib and delanzomibs are reversible third generation oral boronic acid PIs [87]. Carfilzomib obtained FDA approval on the strength of a phase 2 study, showing significant efficacy in refractory/relapsed (RR) MM (overall response rate of 23.7% with median duration of response and median overall survival of 7.8 and 15.6 months in patients who were refractory to bortezomib) [90]. In preclinical studies, oprozomib has demonstrated cytotoxicity in MM in combination with lenalidomide and/or HDAC inhibitor molecules, as well as bone anabolic effects [91,92]. The salinosporamide PI marizomib inhibits all three catalytic subunits of the proteasome and is currently in clinical development in MM (NCT02103335).

### 3.2. Autophagy Inhibitors

Dual inhibition of autophagy and the UPS with hydroxychloroquine (HCQ) in combination with bortezomib, has recently emerged as a potentially useful strategy for overcoming therapeutic resistance to proteasome inhibition [93]. Specifically, a phase I clinical trial reported a very good partial response (VGPR) rate of 14%, when HCQ was used in combination with bortezomib in refractory/relapsed MM (RRMM) (Table 1) [93]. HCQ also demonstrates synergistic activity when used in combination with carfilzomib [94]. Bafilomycin A1, an inhibitor of the late phase of autophagy, has also been reported to potentiate the anti-MM activity of bortezomib by inducing irreparable ER stress [73].

### 3.3. Blocking the Aggresomal Pathway through HDAC6 Inhibition

Panobinostat is a broad-spectrum HDAC inhibitor (HDACi) that has been FDA approved for use in refractory/relapsed MM (RRMM) in combination with bortezomib and dexamethasone based on favorable clinical trial results demonstrating a 35% overall response rate (ORR) [95]. However, non-selective HDAC inhibitors have a narrow therapeutic index, thereby prompting the development of isoform-specific HDAC inhibitors such as ACY-1215, a HDAC6-selective inhibitor. By disrupting aggresome formation, preclinical studies have shown that ACY-1215 synergizes with proteasome inhibition (with bortezomib and carfilzomib) against MM cells [96,97]. A multicenter phase IB clinical trial assessing the efficacy of ACY-1215 in combination with lenalidomide and dexamethasone in RRMM reported an overall response rate (ORR) of 55% [98].

### 3.4. Heat Shock Protein 90 (HSP90) Inhibitors

HSP90 is the most well-studied heat shock protein in MM. HSP90 inhibitors in preclinical development include NVP-HSP990 and TAS-116 [99,100]. TAS-116 in particular has shown promising synergism with bortezomib treatment [100]. Clinically, a phase I/II trial of tanespimycin (HSP90 inhibitor) in combination with bortezomib used in the treatment of RRMM reported a 27% ORR [101]. However, further development of tanespimycin was halted due to patent expiry in 2014 which made it hard to justify further financial investment in the drug [102].

### 3.5. Unfolded Protein Response (UPR) Modulators

Drugs such as tunicamycin, thapsigargin, and brefeldin A are ER stressors that lead to the induction of the UPR. Despite good anti-MM activity and synergism with proteasome inhibitors in vitro, clinical translation has been limited by anticipated toxicities based on in vivo studies [29,103,104,105]. More recent efforts at targeting the UPR have led to the development of IRE1α endoribonuclease domain specific inhibitors such as MKC-3946. By inhibiting Xbp1 splicing, MKC-3946 activates the UPR through the PERK pathway resulting in eIF2α phosphorylation and increased ATF4 and CHOP expression; thereby enhancing ER stress-mediated apoptosis induced by bortezomib [106]. It is worthwhile noting that while the inhibition of Xbp1 splicing in terminally differentiated MM (plasma) cells triggers apoptosis in MM cells, Xbp1 splicing is paradoxically also required for plasma cell maturity which is a key determinant of PI sensitivity (mature plasma cells have greater proteasome workload and are therefore more sensitive to PI) [81]. The HIV protease inhibitor nelfinavir has also been shown to potentiate the anti-MM activity of bortezomib through the induction of the UPR and CHOP-dependent apoptosis [107]. A phase II trial of nelfinavir and bortezomib reported an ORR of 30% in the dose escalation cohort and an ORR of 50% in an exploratory extension cohort comprising patients with both bortezomib-refractory and lenalidomide-resistant MM (SAKK 65/08) [108]. Finally, targeting ERAD through pharmacological inhibition of p97 using Eayarestatin 1 and DBeQ has recently emerged as a means to disrupt intracellular protein metabolism within MM cells [67]. Of note, dual inhibition of p97 and the proteasome induced significant apoptosis in both cell lines and patient-derived MM cells with minor toxicity observed in untransformed, non-secretory control cells [67].

## 4. Exploiting Proteotoxic Stress in Solid Tumors: Triple Negative Breast Cancer (TNBC)

### 4.1. Proteasome Inhibition in TNBC

While proteasome inhibitors have shown significant clinical efficacy in multiple myeloma and mantle-cell lymphoma treatment, their effect against solid tumors such as triple negative breast cancer (TNBC) has been less than encouraging [88,109,110]. TNBC is an aggressive chemoresistant subtype of breast cancer with a poor clinical outcome. It is characterised by lack of expression of the estrogen receptor and the progesterone receptor, and the lack of overexpression of human epidermal growth factor receptor 2 (HER2). Treatments are limited and non-specific, due to its innately treatment-resistant biology. A recent study has revealed that inhibition of both the β5 and β2 sites of the proteasome sensitised triple negative breast cancer cell lines to bortezomib [111]. This suggests that a dual inhibitor may enhance the clinical benefit of bortezomib in solid tumors. Moreover, bortezomib, engineered to be contained in nanoparticles, has shown promise as a drug delivery system for TNBC by overcoming its clinical limitations such as low water solubility [112]. Furthermore, the chemotherapeutic paclitaxel has been shown to regulate the genes involved in the ubiquitin proteasome system in breast cancer. Specifically, paclitaxel treatment resulted in an upregulation of proteasome genes and lead to an accumulation of proteasome subunits alpha 4 and beta 1, 26S ATPases 2 and 6 and 26S non-ATPase 14. Moreover, proteasome inhibition with MG132 following paclitaxel treatment resulted in growth inhibition and apoptosis [113]. 

Clinically, a phase I/II trial has shown that the combination of capecitabine and bortezomib in anthracycline and taxane pre-treated metastatic breast cancer patients was well tolerated [114]. These studies suggest that a combination regime of paclitaxel with proteasome inhibitors is a potential treatment strategy for breast cancer patients. Studies have also suggested that the combination of lapatinib and proteasome inhibitors may be beneficial for patients with a diagnosis of TNBC [115]. Importantly, a case study of a patient with TNBC who had adverse side effects to standard treatment, was reported to have an 11-month progression free survival following bortezomib treatment [116].

Additional in-vitro studies have shown that bortezomib induced significant apoptosis in three TNBC cell lines: HCC-1937, MDA-MB-231, and MDA-MB-468 via the inhibition of CIP2A (cancerous inhibitor of protein phosphatase 2A) [117]. Moreover, bortezomib demonstrated in-vivo cytotoxic activity in HCC-1937 xenografted tumors [117]. Bortezomib is a known substrate of P-glycoprotein, a multidrug efflux transporter that plays a role in resistance to proteasome inhibition by facilitating PIs to be pumped out of cancer cells, and it has been demonstrated that inhibition of the P-glycoprotein using verapamil sensitizes MDA-MB-231 TNBC cells to the proteasome inhibitors MG132, bortezomib and carfilzomib [118,119,120]. Intriguingly, gene silencing studies using small interfering RNA (siRNA) have shown that “basal-like” TNBC cells were dependent on genes implicated in the proteasome (PSMA1, PSMA2, PSMB4) for survival as silencing of these genes resulted in ≤50% viability compared to control siRNA-transfected cells [121]. Consistent with the siRNA screen, basal-like TNBC cells were more sensitive to proteasome inhibitors than other breast cancer types [121], suggesting the potential promise of using proteasome inhibitors as a treatment strategy for TNBC patients.

### 4.2. Cellular Senescence and Proteotoxic Stress: A Double-Edged Sword?

The concept that cells will only divide until they reach a limit of cell division or replicative induced senescence was devised by Hayflick (1961) and is termed the “Hayflick limit” [122]. This process of replicative induced senescence (RIS) occurs due to telomere shortening after every round of cell division. Senescence can also be induced specifically in cancer cells which are exposed to treatment “stressors” such as radiation therapy or chemotherapy. Ideally, chemotherapy induces tumor cell death via apoptosis. Paradoxically, tumor cells can maintain viability in response to chemotherapy, by undergoing alternative fates such as therapeutic-induced-senescence (TIS) [123]. Although senescent cells are metabolically active, they are also proliferatively incompetent [124]. Therefore, chemotherapies such as paclitaxel, which preferentially impact on dividing cells, are less likely to induce apoptotic cell death [125]. Increased expression of p21, p16, and senescence associated β-galactosidase (SA-β-Gal) activity are well-established markers of senescent cells [126,127]. Moreover, senescent cells secrete the senescence associated secretory phenotype (SASP); a secretome known to be associated with cancer promoting phenomenon such as chronic inflammation, angiogenesis, cell proliferation and cell invasion [128]. In addition, the ubiquitin proteasome system has been implicated in the selective degradation of proteins involved in the onset and/or maintenance of senescence in a process known as senescence associated protein degradation (SAPD) [129]. 

Endoplasmic reticulum (ER) stress can occur in the senescent phenotype resulting from the misfolding of proteins. This leads to the activation of the unfolded protein response (UPR) to deal with the resultant misfolded proteins and proteotoxicity. A recent study of the WI-38 model of replicative senescence and stress induced senescence induced by hydrogen peroxide and copper sulfate demonstrated an impairment in the chaperoning mechanisms of the ER and UPR activation [130]. Activation of the UPR may therefore represent a mechanism by which senescent cells can maintain viability and cellular survival in a stressful environment [131]. Another study has shown that WI38 fibroblasts that entered senescence at about 40 cumulative population doublings, had a reduced proteasome function evidenced by increased levels of both oxidised and ubiquitinated proteins, which could be explained by reduced protein expression of the catalytic subunits of the 20S proteasome and subunits of the 19S regulatory complex [132]. Intriguingly, partial inhibition of the proteasome with MG132 or epoxomicin induced a senescence-like phenotype in early-passage WI38 cells, suggesting that loss of proteasome function could be a cause rather than an effect of cellular senescence [132]. Taken together, these studies demonstrate that proteostasis plays a central role in both the induction of cellular senescence and the maintenance of cell viability during senescence.

Exosomes are extracellular vesicles (EVs) that are ~50–150 nm in size. These vesicles are of endocytic origin released into the extracellular space [133], and consist of cytoplasm enclosed with a lipid bilayer [134,135]. EVs are released by multiple cell types and can be found in blood, urine, serum and amniotic fluid [136]. Moreover, therapeutic-induced-senescent (TIS) TNBC cells release significantly more extracellular vesicles (EVs) than control cells [123]. Irradiation induced senescent prostate cancer cells also release more EVs than controls [137]. This suggests that increased EV release could be a potential mechanism conducive to cell survival following cellular stress, in that cells can potentially prevent proteotoxicity and maintain cell proteostasis through the removal of misfolded proteins into the extracellular space using EVs [138,139].

## 5. A Look at What’s on the Horizon

### 5.1. Disrupting the Protein Secretory Pathway to Increase Proteotoxic Stress

The synthesis of secretory or membrane proteins begins at 80S ribosomes attached to the wall of the rough endoplasmic reticulum (ER) [140]. Newly translated proteins made in the ER lumen or membrane are encapsulated within transport vesicles and trafficked to the golgi apparatus where they undergo modifications and folding before being transported to their final destination [140]. Secretory proteins are stored in secretory vesicles juxtaposed to the cell surface membrane where they await appropriate signals for exocytosis [140]. Exocytosis, the fusion of secretory vesicles with the plasma membrane is regulated by intracellular calcium influx and SNARE protein activation [141].

Dual inhibition of protein secretion and degradation has recently emerged as a promising treatment strategy for malignant cells with high protein load. Specifically, by “overloading” MM cells with misfolded proteins, the combination of Sec 61 inhibitors (inhibitor of protein secretion) and carfilzomib or bortezomib, disrupted proteostasis bringing about synergistic cell death [142]. A separate study exploring the use of verapamil (a calcium channel blocker) to interfere with IgG secretion in MM cells, reported that verapamil increased the cytotoxic effect of bortezomib by increasing the accumulation of polyubiquitinated proteins and ER stress signalling within MM cells [143]. Another approach taken to enhance proteotoxic stress in MM cells involves dual inhibition of monoclonal protein trafficking (through the inhibition of the isoprenoid biosynthetic pathway) and protein folding (by targeting HSP90) [144]. Intracellular protein trapping coupled with impaired protein chaperone function resulted in the accumulation of misfolded proteins and the induction of the ER stress, UPR, and ultimately cellular apoptosis [144].

Another strategy to accelerate proteotoxic stress by targeting immunoglobulin secretion in MM was recently explored by Zhou et al. [145]. In this study, a pool of siRNA targeting λ light chains was used to inhibit λ light chain production, which resulted in decreased secretion of intact immunoglobulin G (IgG) λ antibodies (containing paired heavy and light chains) [145]. In the absence of light chains, the accumulation of unstable (unpaired) immunoglobulin heavy chains within the ER resulted in the activation of the terminal UPR and apoptosis [145].

### 5.2. Hijacking E3 Ligases for Specific Target Protein Degradation via the Ubiquitin Proteasome System 

Targeted protein degradation using “degraders” has recently emerged as an attractive and promising approach against currently undruggable (and druggable) targets. Degraders are heterobifunctional small molecules, with two binding motifs separated by a linker that bind to an E3 ubiquitin ligase on one site and a specific protein of interest (POI) on the other site [146]. By redirecting the E3 ligase, degraders facilitate the polyubiquination and subsequent proteasome degradation of specific POIs. The three emerging technologies currently in preclinical development are the (1) PROTAC (PROteolysis Targeting Chimera), (2) Degronimid, and (3) TRIM21 systems [147,148].

#### 5.2.1. PROTAC System

**Figure 2:**
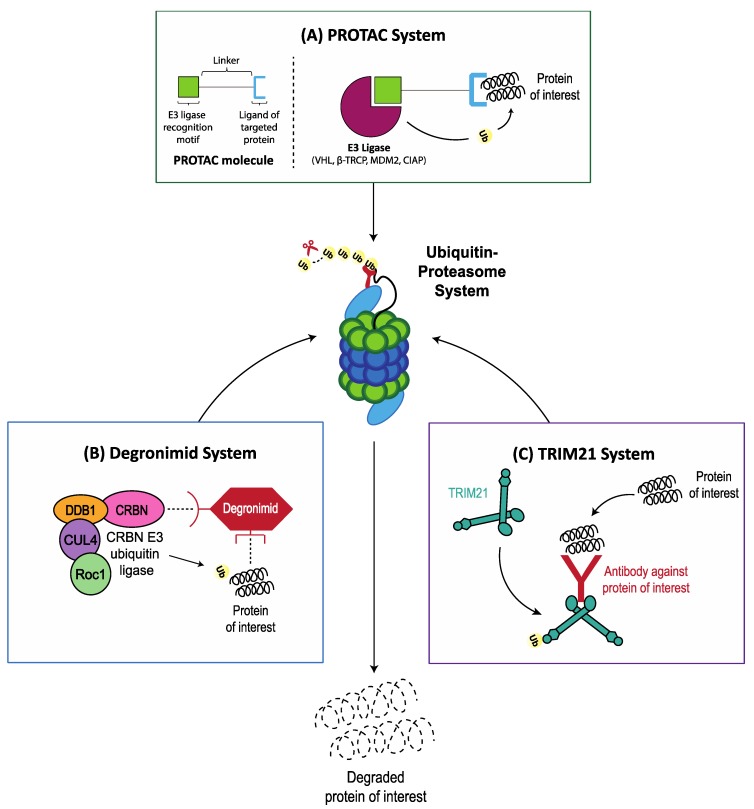
Hijacking the ubiquitin proteasome system (UPR) for targeted protein degradation. Targeted protein degradation has recently emerged as an attractive and promising approach against currently undruggable (and druggable) targets. (**A**) the PROteolysis Targeting Chimera (PROTAC) system: PROTACs are heterobifunctional molecules that serves as a bridge by binding to an E3 ligase on one side and to the protein of interest on the other, thereby facilitating polyubiquitination and proteasome degradation of the protein of interest. (**B**) deronimids are specifically modified immunomodulatory drugs (IMiDs) that recruit the Cereblon E3 ubiquitin ligase to the protein of interest to facilitate target proteasome degradation. (**C**) TRIM21 is an E3 ubiquitin ligase that recognizes and polyubiquitinates antibody-bound substrates by binding with high affinity to the Fc domain of antibodies.

PROTACs are the most well studied degrader technology (Figure 2A). Previous generations of PROTACs were designed to hijack various E3 ligases such as β-TRCP, MDM2 [149], CIAP [150], and von Hippel-Lindau (VHL) [151]. However, these compounds were large and hydrophilic, which affected cell permeability, had low affinity for their targets, and lacked optimal linker geometry [146]. Early PROTACs were therefore very limited in their potency, evidenced by their activity in the low-micromolar range with only partial degradation of POIs [146]. Next-generation PROTACs, on the other hand, are designed with much higher affinities and selectivity for the VHL E3 ligase with due consideration paid to the attachment point, length, and geometry of the linker [146]. As a proof-of-concept, next-gen PROTACs that were developed against serine-threonine kinase RIPK2 had the ability to specifically reduce protein levels by >90% at nanomolar concentrations both in-vitro and in-vivo [152].

#### 5.2.2. Degronimids

In 2014, the discovery that the immunomodulatory drugs (IMiDs) lenalidomide and pomalidomide act as a bridge between the Cereblon E3 ubiquitin ligase and Ikaros/Aiolos to enhance ubiquitination and subsequent proteasome degradation of the latter, brought fresh perspectives to the field of targeted protein degradation [153,154]. By utilizing degronimids (modified IMiDs) to alter (hijack) the activity of Cereblon E3 ubiquitin ligase, the UPS can be manipulated to achieve targeted degradation of proteins within cells (Figure 2B). An example of a degronimid in preclinical development is the phthalimide conjugate dBET1 which specifically and potently degrades BRD4 in human leukemic cells; resulting in rapid and robust apoptosis both in-vitro and in-vivo [147]. 

The challenge in designing effective heterobifunctional degraders that bridge POIs to an E3 ubiquitin ligase is learning how to maximize both selectivity and potency by varying linker composition and length [155]. This design process would have to be optimized for each individual POI, and would require the up-front identification of a target-selective ligand [155]. To circumvent this, Nabet et al. developed the degradation TAG (dTAG) system: a two-step process involving (1) CRISPR/Cas-9 mediated knock-in of an FKBP12^F36V^ protein tag, next to the gene of interest, and (2) the addition of a degrader that bridges the resultant FKBP12^F36V^ fusion protein of interest to the Cereblon E3 ligase [155]. This single generalisable approach allows for the rapid degradation of allele-specific protein chimeras, thereby serving as a powerful tool for target validation and biological investigation in the context of drug discovery.

#### 5.2.3. TRIM21 System

TRIM21 is an E3 ubiquitin ligase that recognizes antibody-bound pathogens by binding with high affinity to the Fc domain of antibodies [156,157]. Clift et al. developed a three-step strategy to repurpose TRIM21 to achieve targeted protein degradation: (1) introduction of exogenous TRIM21, (2) introduction of an antibody against the POI, and (3) TRIM21-mediated polyubiquination of antibody-bound POI and subsequent degradation by the UPS (Figure 2C) [158]. Like the dTAG system, TRIM21 represents a novel method for studying protein function with future therapeutic potential, especially in cancers that are highly susceptible to proteasome overload.

## 6. Conclusions

The modern oncology paradigm hinges on genomic characterisation of tumors to increase our fundamental understanding of cancer biology, thereby facilitating the design of novel therapeutics exploiting various cancer vulnerabilities such as “oncogene addiction”. While this ideal of precision medicine is an elegant approach to targeting cancer, tumor heterogeneity makes targeting multiple continually evolving clonal and genetic abnormalities with the right combination, at appropriate times, and in the correct sequence highly challenging [159]. This is why targeting the consequences of genomic instability, such as blocking proteotoxic stress responses in cancer constitutes an attractive therapeutic strategy that is echoed by the success of proteasome inhibition in MM. In spite of this, most patients inevitably develop clinical resistance to proteasome inhibitors, prompting the development of novel therapies that could potentially synergise with PIs to target other pathways involved in protein quality control in cancer cells. Importantly, preclinical and early clinical trials suggest the potency of combinations, such as PIs with UPR modulators, PIs with HDAC6 inhibitors, and PIs with autophagy inhibitors.

Particularly exciting developments in the space of triple negative breast cancer (TNBC) are reports suggesting that proteasome inhibition may be an effective strategy for reversing chemotherapy-induced senescence; a mechanism that TNBC cells use to maintain viability and cellular survival and acquire cancer chemotherapy resistance. Phase I/II clinical trials provide early evidence supporting the use of a combination regime of proteasome inhibitors with paclitaxel, capecitabine, or lapatinib in the treatment of anthracycline and taxane pre-treated metastatic breast cancer patients.

Finally, by hijacking the ubiquitin proteasome system within cancer cells, emerging technologies aimed at repurposing various E3 ubiquitin ligases towards targeted protein degradation offer a powerful tool for target validation and biological investigation in the context of drug discovery. In conclusion, our increasing understanding of the over-reliance of cancer cells on proteostasis pathways has led to the ongoing development of various distinct therapeutic strategies with the common goal of exacerbating proteotoxic stress to cause irreparable damage and cancer cell apoptosis.

## Figures and Tables

**Table 1:** Therapies targeting protein handling pathways in multiple myeloma (MM).

Drug Class	Drug Name	Mechanism of Action	Study Design	Status
**PI**	Bortezomib	Proteasome inhibition; Apoptosis via caspase 8/9; UPR apoptotic response	Single use approval	FDA approved
	Carfilzomib	Irreversible proteasome inhibition	Single use approval; Combination treatment DEX and/or LEN	FDA approved
	Ixazomib	Oral Proteasome inhibitor	Combination treatment with LEN and DEX	FDA approved
	Oprozomib	Proteasome inhibition; Apoptosis via caspase 8/9	Single agent	Phase IB/II
	Marizomib	Pan-Proteasome inhibition; Apoptosis via caspase 8/9; UPR apoptotic response	Single agent	Phase I
**UPR Modulators**	MKC-3946	Inhibition of XBP1 splicing by IRE1α endoribonuclease domain inhibition	Combination treatment with bortezomib	Preclinical
	Nelfinavir	Activation of PERK apoptotic pathway; Upregulation of CHOP; Inhibition of AKT phosphorylation	Combination treatment with bortezomib	Preclinical
			Combination treatment with bortezomib in R/R and progressive MM	Phase I
**HDACi**	Panobinostat	Broad spectrum inhibitor of HDAC leading to aggresome disruption; Apoptosis via caspase 8/9; UPR apoptotic response	Combination treatment with bortezomib and DEX (where 2 or more treatment options have been used prior)	FDA approved
	ACY-1215 (Ricolinstat)	Selective inhibition of HDAC6 leading to aggresome disruption; Apoptosis via caspase 8/9; UPR apoptotic response	Combination treatment with bortezomib	Preclinical
			Combination treatment with carfilzomib	Preclinical
			Combination treatment with LEN and DEX	Phase IB
**Autophagy Inhibitors**	Hydroxy-chloroquine	Inhibition of autophagy by increased lysosomal pH	Combination treatment with bortezomib R/R MM	Phase 1
			Combination treatment with carfilzomib	Preclinical
	Bafilomycin A1	Inhibition of autophagy by prevention of autophagosome/lysosome fusion	Combination treatment with bortezomib	Preclinical
**HSP Inhibitors**	NVP-HSP990	HSP90 inhibitor; Disruption of AKT, JAK/STAT pathways	Single agent	Preclinical
	TAS-116	HSP90 inhibitor Induction of apoptosis Disruption of AKT & ERK	Single agent; Combination treatment with bortezomib	Preclinical
	Tanespimycin	HSP90 inhibitor Induction UPR	Combination treatment with bortezomib	Phase I/II
